# Gait analysis in adolescent idiopathic scoliosis walking with Boston brace

**DOI:** 10.1186/1748-7161-9-S1-O24

**Published:** 2014-12-04

**Authors:** Mohammad Taghi Karimi, Mahsa Kavyani, Mohammad Reza Etemadifar

**Affiliations:** 1 Musculoskeletal Research Center, Isfahan University of Medical sciences, Isfahan, Iran; 2 Orthopedic Surgery Department, School of Medicine, Isfahan University of Medical sciences, Isfahan, Iran

**Keywords:** Scoliosis, kinematic, range of motion

## Background

Adolescent idiopathic scoliosis (AIS) can affect spine mobility and gait mechanisms. In some of the related science research it is showed that the kinematic differences in the spine, pelvis and lower limb may contribute to the causation and progression of idiopathic scoliosis. Various treatment methods have being used for scoliosis, however using brace is a commonly used method in this regard. Nowadays little is known about the effects of bracing on gait biomechanics in scoliotic patients. The aim of this investigation was to identify the immediate effects of bracing on improvement of asymmetries in lower limb kinematics and pelvic and back movements during level walking in scoliotic subject.

## Method

Twenty subjects (10 healthy subjects and 10 AIS with thoracolumbar/lumbar curve) were recruited in this study. Gait analysis was assessed using a three-dimensional motion analysis and a Kistler force plate. Scoliotic patients were assessed with and without Boston brace. Spatiotemporal gait parameters and kinematic parameters of the thorax, pelvis, hip, knee and ankle joints were the parameters used in this study.

## Result

Bracing had no significant effect on body segment excursion of ankle, knee and hip joints; however pelvis and hip motions were significantly reduced in all AIS patients.

## Conclusion

The use of orthosis seems to improve the symmetry of motion of right and left sides in hip and pelvic. However it reduces the motions of these joint in scoliotic subjects.

